# Spatial Distribution and Climate Warming Impact on *Abies kawakamii* Forest on a Subtropical Island

**DOI:** 10.3390/plants11101346

**Published:** 2022-05-19

**Authors:** Ching-An Chiu, Hsy-Yu Tzeng, Cheng-Tao Lin, Kun-Cheng Chang, Min-Chun Liao

**Affiliations:** 1 Experimental Forest, National Chung Hsing University, No. 145 Xingda Rd., Taichung City 40227, Taiwan; cachiu@nchu.edu.tw; 2 Department of Forestry, National Chung Hsing University, No. 145 Xingda Rd., Taichung City 40227, Taiwan; 3 Department of Biological Resources, National Chiayi University, No. 300 Syuefu Rd., Chiayi City 60004, Taiwan; mutolisp@mail.ncyu.edu.tw; 4 Department of Forestry and Natural Resources, National Chiayi University, No. 300 Syuefu Rd., Chiayi City 60004, Taiwan; kcchang@mail.ncyu.edu.tw; 5 Botanical Garden Division, Taiwan Forestry Research Institute, No. 53 Nanhai Rd., Zhongzheng District, Taipei City 10066, Taiwan

**Keywords:** *Abies kawakamii*, endemic species, species distribution modeling, Taiwan, climate warming

## Abstract

Species distribution modeling (SDM) is currently the primary tool for predicting suitable habitats for species. In this study, we used *Abies kawakamii*, a species endemic to Taiwan. Being the only *Abies* species distributed in high mountains, it acts as an ecological indicator on the subtropical island. We analyzed a vegetation map derived from remote sensing and ground surveys using SDM. The actual distribution of *A. kawakamii* in Taiwan has a total area of 16,857 ha distributed at an altitude of 2700–3600 m, and it often forms a monodominant forest at 3100–3600 m with the higher altitude edge as a forest line. Exploring the potential distribution of *A. kawakamii* through MaxEnt showed that the suitable habitat was 73,151 ha under the current climate. Under the scenarios of temperature increases of 0.5, 1.0, 1.5, and 2.0 °C, suitable habitat for *A. kawakamii* will gradually decrease to 70.2%, 47.1%, 30.2%, and 10.0% of this area, respectively, indicating that *A. kawakamii* will greatly decline under these climate warming scenarios. Fire burning disturbance may be the most significant damage to *A. kawakamii* at present. Although *A. kawakamii* has been protected by conservation areas and its natural regeneration is in good condition, it rarely has the opportunity to migrate upwards during climate warming. We suggest that in the future, research on the natural regeneration and artificial restoration of *A. kawakamii* should be emphasized, especially in the forest line ecotone.

## 1. Introduction

Species distribution modeling (SDM) is currently the primary tool for predicting suitable habitats for species [1,2,3,4]. SDM links the ecological theory of species–environment relationships with statistical learning methods and geospatial data to understand and predict species distributions and habitats [5]. It is widely used in biogeography, biological conservation, and environmental change research [6,7,8]. In recent years, the number of SDM-related studies has increased in number each year [9,10]. SDM is a powerful tool to support forest management, and it can be used to forecast climate change impacts on forests [11]. To date, many different methods have been developed to predict species occurrences based on environmental characteristics [11,12,13]. Among these SDM methods, MaxEnt (maximum entropy) [14,15,16] is the most commonly used. Many studies have used MaxEnt to explore habitat suitability and climate change [17,18]. Compared with the more complex ensemble SDM, the MaxEnt model’s performance is comparable to the ensemble approach, and MaxEnt has the advantages of reduced calculation time and simplicity [19].

The genus *Abies* is composed of approximately 50 species globally, which are distributed in the subalpine to alpine regions of Europe, Asia, North America, Central America, and the northernmost part of Africa, mostly in cold and temperate climates in the Northern Hemisphere [20,21]. Only four *Abies* species (i.e., *Abies kawakamii* (Hayata) T.Itô, *A. webbiana* (Wall. ex D.Don) Lindl., *A. religiosa* (Kunth) Schltdl. & Cham., and *A. fansipanensis* Q.P.Xiang) are distributed in subtropical regions, and all of them are ecological indicators of the genus [20,21,22]. Among them, *A. kawakamii* is an endemic and relic tree species that composes monodominant forest and builds forest lines in high mountain areas in subtropical Taiwan [23,24,25,26,27]. It plays an essential role in the subalpine ecosystem and presents a unique forest landscape [28,29]. Plants at high altitudes are susceptible to climate change [30,31] and rarely have the opportunity to migrate upwards under warming conditions [32,33,34]. *Abies* tree species that grow on the island’s high mountains, such as *A. koreana* [35,36,37], may face a more severe decline. Unfortunately, both past observations and future predictions show a clear warming trend for Taiwan [38,39,40], and it is necessary to understand the spatial distribution of *A. kawakamii* in the high mountain areas of this subtropical island. This study analyzed a vegetation map derived from remote sensing and ground surveys of *A. kawakamii* using SDM. Evaluating actual and potential spatial distribution, we predict the future trend of the distribution area of *A. kawakamii* under climate warming scenarios.

## 2. Results

### 2.1. Current Actual Distribution of A. kawakamii

**Figure 1:**
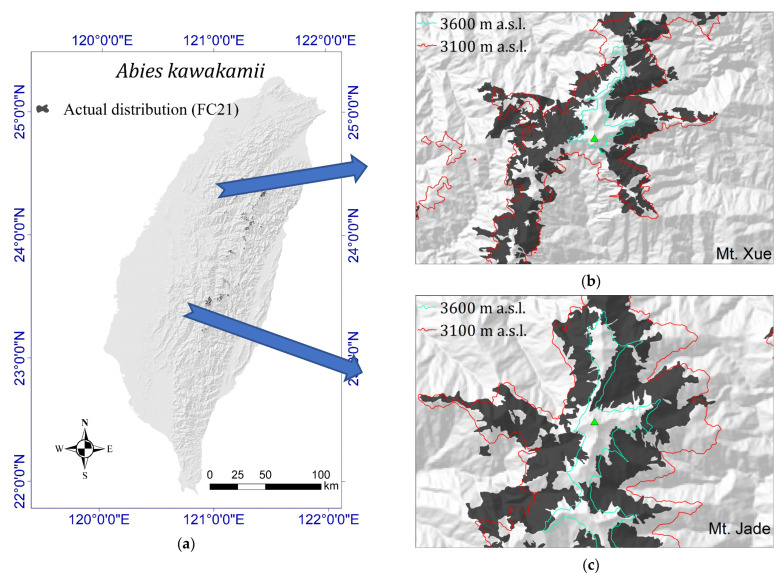
Actual distribution of *Abies kawakamii* (FC21 formation, black polygon) in Taiwan. Red line: 3100 m contour; blue line: 3600 m contour. (**a**) Taiwan island, (**b**) Mount Xue, and (**c**) Mount Jade.

Table 1 reveals the actual area of *A. kawakamii* in each 100 m altitude zone. The current actual distribution of *A. kawakamii* was extracted from the FC21 polygon (*A. kawakamii* formation represented as a black polygon in Figure 1) of the TVDIM vegetation map [41]. The results show that the total area of the actual area distribution of *A. kawakamii* is 16,857 ha, which is only 0.47% of the total area on the island of Taiwan (Figure 1a). No particular difference was detected in the distribution of *A. kawakamii* in different directions (Figure 1). The actual distribution of *A. kawakamii* is mainly concentrated on high mountains such as Mount Jade (Figure 1b) and Mount Xue (Figure 1c).

### 2.2. Potential Distribution Modeling Using MaxEnt

After calculating the correlation coefficients of 21 pre-selected environmental variables, for the 8 environmental variables selected and used in MaxEnt (Table 2), EWI occupied absolute importance, which means that the distribution of *A. kawakamii* is mainly controlled by altitude or a more precise thermal index.

**Figure 2:**
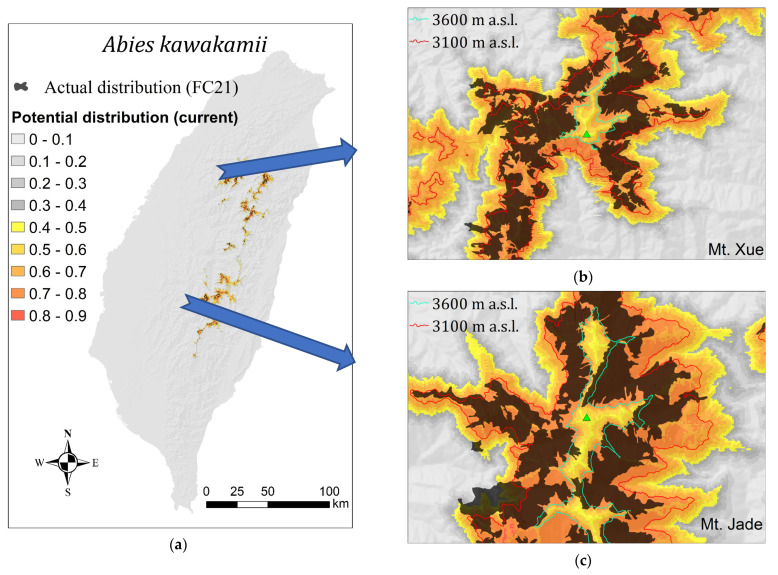
Potential (occurrence probability under the current climate, orange tone) and actual (FC21 formation, black polygon) distribution of *Abies kawakamii* forest. (**a**) Taiwan island, (**b**) Mount Xue, and (**c**) Mount Jade.

The AUC value of our model was 0.9628, which indicates excellent accuracy [42]. The potential distribution or the occurrence probability of *A. kawakamii* simulated by MaxEnt is shown in the orange tone in Figure 2, and its Cloglog log value ranged from 0.0000 to 0.8580. Two commonly used thresholds of species presence/absence [43,44,45] were calculated. Threshold 1 (equal training sensitivity and specificity = 0.4046) was more reasonable than threshold 2 (maximum training sensitivity plus specificity = 0.0972). Therefore, we used a threshold of 0.4046 to determine the presence or absence of *A. kawakamii* (Figure 2).

**Figure 3:**
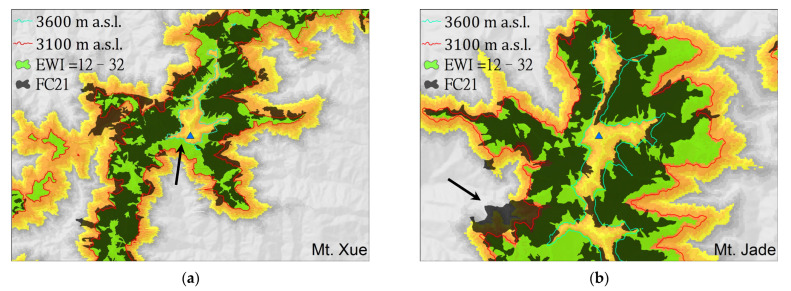
Predictive distribution of *Abies kawakamii*, using EWI = 12–32 (green polygon), overlapping its actual (FC21, block polygon) and potential (occurrence probability, orange tone) distribution. (**a**) Mt. Xue and (**b**) Mt. Jade.

Figure 3 shows the results of predicting the distribution of *A. kawakamii* using 3100–3600 m contours [23,24] and EWI (effective warmth index) = 12–32 [24,46], and overlapping its actual and potential distributions.

**Table 2:** Percent contribution and permutation importance of the eight variables used in MaxEnt.

Variables	Description and Reference	Percent Contribution	Permutation Importance
EWI	Effective warmth index [46]	97.6	99.6
PER	Potential evapotranspiration ratio [47]	1.0	0.0
bio15	Precipitation seasonality [48]	0.4	0.0
WLS	Whole light sky space [49]	0.4	0.1
PWR	Ratio of winter half-year precipitation to Bio12 [23]	0.2	0.1
bio12	Annual precipitation [48]	0.2	0.0
bio4	Precipitation of driest month [48]	0.1	0.0
bio9	Precipitation of coldest quarter [48]	0.0	0.1

### 2.3. Potential Distribution under Climate Warming Scenarios

**Figure 4:**
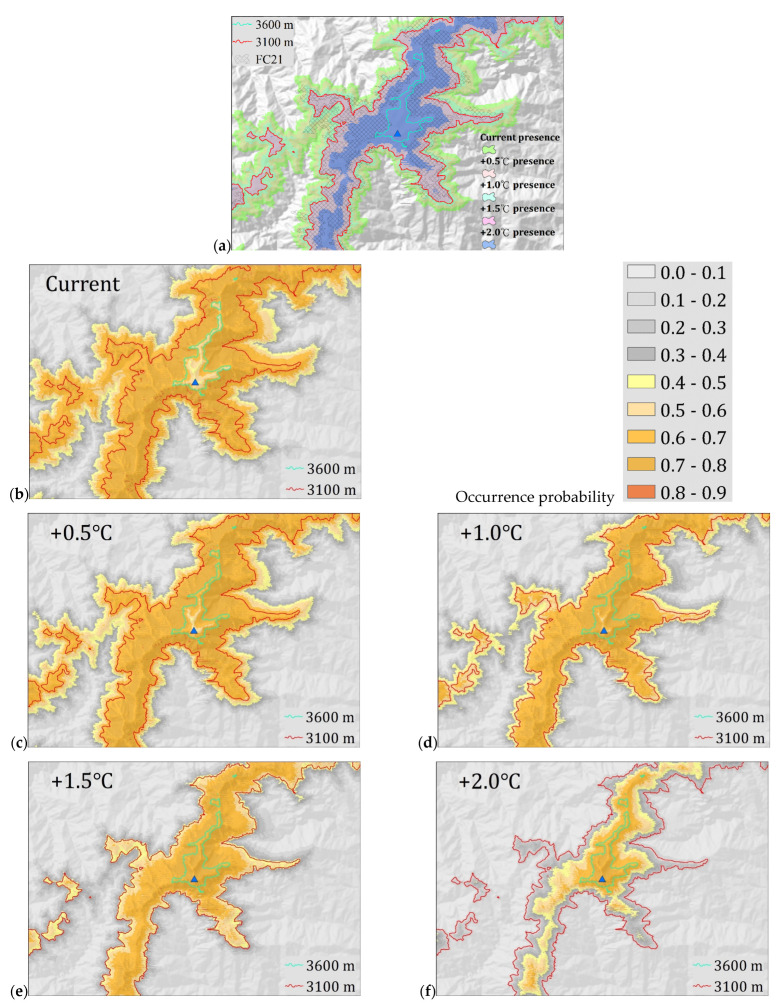
Changes in suitable habitat areas of *A. kawakamii* on Mount Xue (**a**) under different climate warming scenarios, (**b**) current climate, (**c**) under the +0.5 °C scenario, (**d**) under the +1.0 °C scenario, (**e**) under the +1.5 °C scenario, and (**f**) under the +2.0 °C scenario.

*A. kawakamii* will gradually lose suitable habitats at lower altitudes under increasing temperatures of 0.5, 1.0, 1.5, and 2.0 °C. Using equal training sensitivity and specificity as the threshold to convert the occurrence probability value to presence–absence binary data, when the temperature increased by 0.5, 1.0, 1.5, and 2.0 °C, the presence areas of *A. kawakamii* were 51,323, 34,476, 22,106, and 7338 ha, respectively (Table 3); that is, the suitable habitats of *A. kawakamii* will gradually decrease to 70.2%, 47.1%, 30.2%, and 10.0% of the current area. This illustrates the loss and gain of *A. kawakamii* habitat areas under the +0.5~+2.0 °C climate warming scenario. Overall, *A. kawakamii* is migrating and shrinking to high-altitude areas as the temperature rises. In other words, it loses its original habitats in lower places and gains new habitats in higher places, and the area lost is much larger than the area gained. Table 4 further explains the loss and gain changes in Figure 4. We defined Cloglog ± 0.1–0.3 as a low loss or gain change, Cloglog ± 0.3–0.5 as a medium change, and Cloglog ± 0.5–0.7 as a high change.

**Table 3:** Summary of the spatial distribution layers of *A. kawakamii*.

Layer	Area (ha)	Habitat	Meaning and Reference
Actual distribution	16,857	Actual	Combining the ground survey of existing vegetation and the interpretation of remote sensing images to map the current actual distribution polygons of *A. kawakamii*-dominant forests [41]
Range of 3100–3600 m contours	25,571	Potential	The altitude range of *A. kawakamii* forests observed in the field [24,50]
Range of EWI (effective warmth index) = 12–32	20,922	Potential	Use of the more ecologically significant thermal index (relative to the altitude) to estimate the temperature niche of the *A. kawakamii* forest belt [46]
Range of WI (warmth index) = 12–36	20,989	Potential	Use of the more ecologically significant thermal index (relative to the altitude) to estimate the temperature niche of the *A. kawakamii* forest belt [50]
Presence range of potential distribution for the current climate	73,151	Potential	A species-suitable index value [51] or predicted probability of occurrence [16] of *A. kawakamii* individuals is higher than the presence threshold
Presence range of potential distribution for the +0.5 °C scenario	51,323	Potential	Predicted distribution of *A. kawakamii* by MaxEnt under the +0.5 °C scenario [52]
Presence range of potential distribution for the +1.0 °C scenario	34,476	Potential	Predicted distribution of *A. kawakamii* by MaxEnt under the +1.0 °C scenario [52]
Presence range of potential distribution for the +1.5 °C scenario	22,106	Potential	Predicted distribution of *A. kawakamii* by MaxEnt under the +1.5 °C scenario [52]
Presence range of potential distribution for the +2.0 °C scenario	7338	Potential	Predicted distribution of *A. kawakamii* by MaxEnt under the +2.0 °C scenario [52]

## 3. Discussion

### 3.1. Current Actual Distribution of A. kawakamii

**Figure 5:**
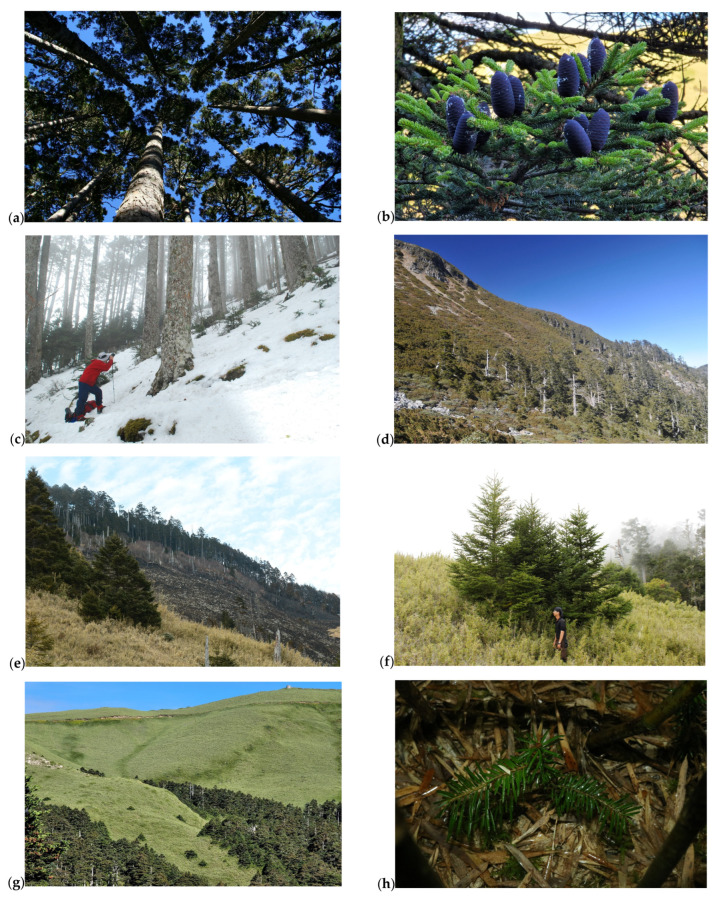
*Abies kawakamii* forests in Taiwan: (**a**) monodominant species forest, (**b**) cone of *A. kawakamii*, (**c**) gap regeneration (on the left), (**d**) forest line, (**e**) fire disturbance, (**f**) regeneration after fire disturbance, (**g**) topographic sheltering, and (**h**) seedling in understory, *Yushania niitakayamensis*.

*A. kawakamii* grows in the altitude zone of 2300–3800 m asl (Table 1), and the total actual area of *A. kawakamii* is 16,857 ha, which is less than the 20,000 ha claimed by the Taiwan Forest Bureau in previous investigations [42]. In the past, the low-altitude limit of *A. kawakamii* has been overestimated [20,50,53]. The area of *A. kawakamii* forest appearing in the 2700–3100 m altitude zone is 39.30%, and it is 57.0% in the 3100–3600 m zone and 96.3% in the 2700–3600 m zone. Many reports [24,26,50] have mentioned that *A. kawakamii* is mainly distributed at an altitude of 3100–3600 m asl. Therefore, we overlapped these two contour lines, and Figure 1b,c show that most *A. kawakamii* fall within this altitude range. Through field observation and investigation [25], it was determined that *A. kawakamii* exist as monodominant forests (Figure 5a) at 3100–3600 m, often mixing with *Tsuga chinensis* below 3100 m asl and mixing with *Juniperus morrisonicola* to form the forest line (Figure 5d) above 3600 m. The current actual distribution of *A. kawakamii* is located in the region of latitude 23.05120–24.47351 N and longitude 120.86114–121.49303 E (Figure 1a). Compared with *A. fansipanensis*, which is distributed in the southernmost limits of Eurasian *Abies* species and only occurs on mountains of 2900–3100 m in northern Vietnam (latitude 22°18′ N and longitude 103°48′ E [22]), *A. kawakamii* is the second southernmost species in Asia. Both *A. fansipanensis* and *A. kawakamii* are isolated from the other Asian *Abies* in distribution and confined to the alpine range.

### 3.2. Potential Distribution Modeling by MaxEnt

Out of 21 pre-selected environmental variables, 13 variables were deleted in order to reduce redundancy and collinearity between variables [54] and to consider their contribution and ecological significance [10,55]. The AUC value of modeling *A. kawakamii* potential distribution by Maxent was 0.9628, which uses eight important environmental variables (Table 2) and indicates excellent accuracy [56].

#### 3.2.1. Range of EWI = 12–32 vs. Actual Distribution of *A. kawakamii*

Su [50] used the warmth index (WI = 12–36) to define the *A. kawakamii* forest belt (with an area of 20,989 ha; in Table 3), which is widely used in Taiwan ecological fields. Meanwhile, Chiu et al. [46] further associated the temperature sum with thermal seasonality to propose the effective warmth index (EWI), and EWI = 12–32 can slightly better fit the *A. kawakamii* forest belt (area of 20,922 ha), as shown in Figure 3. When converting altitude into the thermal index, 3100–3600 m is approximately equivalent to EWI 12–32 (Figure 3). In order to cover the actual distribution of *A. kawakamii*, the lower limit of EWI should be further revised in the future.

#### 3.2.2. Potential vs. Actual Distribution of *A. kawakamii*

Most research on the spatial distribution of plants is based on the equilibrium assumption; that is, analyzing the natural distribution of species to assess the actual living needs of the species [7]. However, due to the facts of chance, isolation, fundamental niche, realized niche [4,57], etc., the actual distribution of plants is often smaller than the potentially suitable distribution area. Thus, Figure 2 and Table 3 clearly show that the potential distribution area of *A. kawakamii* (73,151 ha; orange tone in Figure 2) is larger than the actual distribution area of *A. kawakamii* (16,857 ha; black polygon in Figure 2). As an example, in Figure 3a, the arrow indicates a suitable habitat for *A. kawakamii*, but they do not currently exist there due to the fact of past fire disturbances.

#### 3.2.3. Distributions of Dominant Stands vs. Individuals of *A. kawakamii*

In Table 3, we use *A. kawakamii* individual, *A. kawakamii*-dominant forest, and *A. kawakamii* forest belt to represent the species growing in scattered individuals, sometimes isolated [16,51], in forest formations [24], and in the altitudinal vegetation belt [46,50], respectively. When a species forms a dominant forest, it is usually within its optimum ecological amplitude, and individual plants sometimes extend to the two ends of the environmental amplitude [58]. Our results clearly show that the present area of the simulated *A. kawakamii* individual (73,151 ha; Table 3) is wider than that of the *A. kawakamii*-dominant forest (16,857 ha; actual) or the *A. kawakamii* forest belt (20,922 or 20,989 ha predicted by EWI or WI).

#### 3.2.4. The Influence of Non-Climatic Factors on the Distribution of *A. kawakamii*

Although the climatic climax theory can explain most or large-scale plant distributions, the interaction of factors such as soil, topography, and interspecies competition will modify the actual distribution of *A. kawakamii*. Therefore, the actual distribution of *A. kawakamii* does not appear in the potentially suitable areas near the tops of Mount Xue and Mount Jade (Figure 2b,c), where wind and rock avalanches are strong. On the other hand, the distribution of actual *A. kawakamii* in places with sheltered wind and good soil conditions may exceed its potential range, as indicated by the arrow at the bottom left in Figure 3b.

### 3.3. Potential Distribution under Climate Warming Scenarios

**Figure 6:**
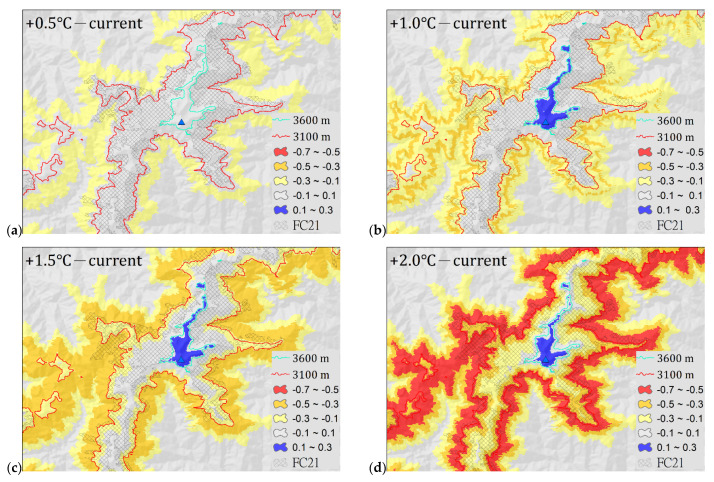
Loss (warm color) and gain (blue) of *A. kawakamii* habitat areas on Mount Xue under the +0.5~+2.0 °C warming scenarios: (**a**) +0.5, (**b**) +1.0, (**c**) +1.5, and (**d**) +2.0 °C.

As can be seen from the overlapped map in Figure 4, *A. kawakamii* shrinks in distribution area under warming scenarios and shows a trend of gradual migration to higher altitudes. The decline in area and upward migration of *A. kawakamii* affected by climate warming are consistent with other *Abies* species or alpine and subalpine plants [35,36,59,60]. The potential distributions of *A. kawakamii* in the +0.5~+2.0 °C warming situations show different degrees of losses of 35,368–71,178 ha and gains of 276–580 ha (Table 4). The loss and gain of these potential distributions mean the upward movement of *A. kawakamii* toward suitable habitats. The range gained at +1.0 °C is not much different from that at +1.5 and +2.0 °C, because the only place where *A. kawakamii* can migrate upward is to the area above 3600 m asl. In addition to the aforementioned substantial decline in the potential habitats of *A. kawakamii* (Figure 4), its current actual distribution is also highly precarious. Due to the *Abies* sensitivity to warming [35,61], the loss of current actual habitats of *A. kawakamii* is likely to occur. Figure 6 reveals that the actual distributions of *A. kawakamii* in the +0.5~+2.0 °C warming scenarios show different degrees of losses of 3991–16,220 ha (also, see Table 4). With the time lag between warming and plant migration [62] and topographic disintegration and interspecies competition when up-shifting [63] (see the *A. kawakamii* forest line in Figure 5d), the gaining of new habitat (Figure 6) by *A. kawakamii* will be quite difficult; in particular, when the temperature rises by 2.0 °C, *A. kawakamii* will have lost 16,220 ha, leaving only 637 ha of habitat. That is, *A. kawakamii* will have lost almost all of its original habitat areas. These few opportunities to migrate to higher altitudes are a common predicament for alpine and subalpine plants [32,33,34,54] and for a variety of Asian subtropical and temperate *Abies* species [35,36].

### 3.4. Conservation and Challenges in the Future

In terms of the in situ conservation of *A. kawakamii*, it is basically only necessary to pay attention to fire disturbance. At present, almost all *A. kawakamii* are protected by national parks and wildlife refuges and have natural regeneration in monodominant forests (Figure 5c) and forest margins (Figure 5f), indicating that the in situ conservation of *A. kawakamii* is good. The most serious damage to *A. kawakamii* in recent years has been caused by man-made fires (Figure 5e), so it is necessary to strengthen fire prevention awareness. No serious pests, diseases, or ungulate animals have been found to harm *A. kawakamii* regeneration [64,65,66,67].

However, the sustainability of *A. kawakamii* is full of crises in the future. Our results point out that both the actual and potential *A. kawakamii* distribution will severely decline in the context of climate warming. In terms of ex situ conservation strategies, *A. kawakamii* seed storage is feasible. *Abies* species are classified as true orthodox seeds [68]. The Taiwan Forest Bureau (TFB) and Taiwan Forest Research Institute (TFRI) currently have a long-term store of *A. kawakamii* seeds. However, seeds for the preservation of marginal and genetically distinct *A. kawakamii* populations should be emphasized, and more biotechnology means have been proposed [69]. Due to the fact that *A. kawakamii* is limited to the highest mountain areas, the escape space available for migration is extremely limited. *A. kawakamii* that has uniform genetic diversity, low differentiation, low numbers of population-specific haplotypes, and neutral evolution characterizes contemporary refuge populations [70]. More is needed than just the ex situ planting that was recommended by Shao et al. [36]. Due to the various obstacles near the *A. kawakamii* forest line, such as topographic disintegration (the grey rubble in Figure 5d), interspecies competition when up-shifting [63], migration speed [59], and warming affecting seedling establishment, relevant research on *A. kawakamii* in the nursery and afforestation settings, suitable habitat availability, colonization, and migration will be necessary. Future climate warming could lead to tree-line advances if viable seeds and suitable substrates for recruitment are available [71]. Based on the fact that *A. kawakamii* is promoted by microclimate and topographic sheltering [72] (Figure 5g) and seedlings appear under *Yushania niitakayamensis* in the transition zone (Figure 5h), we suggest that long-term ecological monitoring programs and regeneration trials of *A. kawakamii* should be carried out in the valleys close to the forest line.

## 4. Materials and Methods

### 4.1. Study Area and Target Species

Taiwan is a mountain island in the continental shelf of East Asia (Figure 1). Its total area is approximately 35,889 km^2^. The highest peak is Mount Jade (3952 m asl), and the second highest is Mount Xue (3886 m asl). There are 268 high mountains above 3000 m asl, which is rare globally. The climate is dominated by the East Asian monsoon, with annual rainfall ranging from 1023 to 4880 mm and the annual average temperature ranging from 4.0 to 25.0 °C [73]. It has alternating winter and summer monsoons, and steep and complex terrain forms diverse plant habitats [50]. During the Ice Age, Taiwan became a refuge for many plants when a large land bridge extended from eastern China to Taiwan. Our target tree species, *A. kawakamii*, migrated from the Eurasian continent to Taiwan island through the Taiwan Strait [22].

Our target species, *A. kawakamii*, is a coniferous relic tree endemic in the high-altitude mountains of Taiwan [50]. It is the only *Abies* tree species that grows on subtropical islands and is close to the southernmost limit of *Abies* species [21,22]. In Taiwan, *A. kawakamii* exists in high-altitude areas, and it is almost a pure forest type (Figure 5a) with good fruiting conditions (Figure 5b). The *A. kawakamii* of today have good natural regeneration in gaps (Figure 5c). At approximately 3600 m asl, *A. kawakamii* has reached the upper limit of its distribution due to the facts of topography, geology, wind, and competition from krummholz species to form a forest line (Figure 5d).

In the past, the understanding of *A. kawakamii* spatial distribution was limited. There were descriptive reports of *A. kawakamii* altitude ranges such as 2400–3600 [20], 2800–3500 [53], and 3100–3600 m [24,50]. Until the completion of the Taiwan Vegetation Diversity Inventory and Mapping Project (TVDIM) [41], a vegetation map of *A. kawakamii* formation (FC21 polygon) was not available, and there is still no detailed analysis of its spatial distribution characteristics. The TVDIM project focused on the mapping of the existing natural vegetation. We extracted the FC21 polygon of the TVDIM vegetation map to further explore the actual distribution of *A. kawakamii* and its relationship with the environment.

### 4.2. SDM Calibration

In brief, SDM consists of three parts [10,74], namely, a dependent variable (species occurrence data), explanatory variables (environmental predictors), and an algorithm or function for representing species–environment relationships (modeling methods). In this article, we used a standard and robust SDM method for presence-only species data [19,75]—MaxEnt—to predict the suitable or potential distribution of *A. kawakamii*. The effectiveness of MaxEnt modeling depends on the appropriate sample sizes and prevalence of species occurrence points [76,77], and on the identification of environmental variables that can explain the species distribution [78,79].

The sources of species occurrence data of *A. kawakamii* included the following: (1) Individual coordinate records from our field survey, all herbariums, and the GBIF database [80]. After filtering for duplicate points and unreasonable coordinates (such as those located in the ocean), a total of 230 points of *A. kawakamii* were used for SDM. (2) Points extracted from the National Vegetation Diversity Inventory and Mapping Project (NVDIMP) database [41] at a fixed distance of 500 m were also used. In this way, 683 occurrence points of *A. kawakamii* were obtained. The 230 points of the former set were extremely unevenly distributed, and most of them were near hiking trails. Therefore, we selected the 683 evenly distributed occurrence points of the latter set as the dependent variable of SDM.

We evaluated a comprehensive set of 62 environmental variables (Table S1), and their consistent spatial resolution was a 40 m grid [10]. Based on our prior knowledge [80], we preselected 21 environmental variables that may affect the distribution of *A. kawakamii*. In order to reduce the redundancy and collinearity of variables [32,54], we adapted the synthetic strategy for integrating the correlation coefficient, contribution level, and expert choice of predictors to select the suitable environmental variables [10]. In addition, Taiwan shows a clear warming trend (1.0–1.4 °C/100 years) and no evidence supporting the possibility of precipitation changes [38]. Thus, we simulated the environment of climate warming with temperature increases of 0.5, 1.0, 1.5, and 2.0 °C and calculated the relevant climate variables in the four scenarios. Currently available high-resolution layers of global climate warming scenarios mainly include three databases: (1) CliMond [81,82] with a maximum resolution of 30 s, (2) WorldClim [51,83] with a maximum resolution of 10 s, and (3) CHELSA [84], although the spatial resolution of this database is too low for *A. kawakamii*. Therefore, in this study, a temperature increase of 2 °C was used as the climate warming scenario, and the 40 m spatial grid layer with temperature increases of 0.5, 1.0, 1.5, and 2.0 °C was recalculated for the environmental variables of MaxEnt. These layers were used as predictors of the influence of the warming scenario on *A. kawakamii*. MaxEnt [16,73,85] was adopted to predict the suitable or potential distribution of *A. kawakamii* in current and warming environments. The operating parameters were random 25-fold cross-validation, 5000 maximum iterations, and in the Cloglog output format with other default settings.

### 4.3. Accuracy Evaluation

The area under the receiver operating characteristic curve (AUC) [86] was used to evaluate the accuracy of MaxEnt. AUC measures the model’s ability to correctly classify a species as present or absent [87]. AUC values range between 0 and 1, with maximum accuracy achieved with values of 1, accuracy no better than random with values of 0.5, and values of <0.5 indicating performance worse than random [85]. The rough guide for classifying MaxEnt modeling accuracy [42] is excellent (AUC > 0.9), good (AUC = 0.8–0.9), average (AUC = 0.7–0.8), poor (AUC = 0.6–0.7), and insufficient (AUC = 0.5–0.6).

### 4.4. Threshold of Species Presence/Absence

MaxEnt produces continuous predictions, namely a Cloglog output, which can be regarded as a species-suitable index value [51] or predicted probability of presence [16]. There are several thresholds, including minimum training presence, 10 percentile training presence, equal training sensitivity and specificity, maximum training sensitivity plus specificity, equate entropy of threshold and original distributions, balance training omission, predicted area, and threshold value, to convert the continuous Cloglog suitability scores predictions of MaxEnt to species presence or absence [16]. The specific thresholds can convert the continuous probability to a binary (presence/absence) map that is easier to apply to species conservation and climate change impacts [88]. In this article, we adopted the recommendations of several reports [43,47,48,53] to use the equal training sensitivity and specificity and the maximum training sensitivity plus specificity as the threshold to reclassify the continuous Cloglog as the presence/absence map of *A. kawakamii*.

## 5. Conclusions

Taiwan is the only habitat of the relic species *A. kawakamii* globally. The detailed distribution and the impact of climate warming on *A. kawakamii* have been unclear in the past. This article explored the actual distribution of *A. kawakamii*. It is concentrated at an altitude of 2700–3600 m asl, covering only 16,857 ha. Its upper edge forms the forest line. In climate warming, the potential habitats of *A. kawakamii* will decline significantly, especially when the temperature rises by 2 °C, it will lose 97.3% of its area. Today, *A. kawakamii* is mainly protected by conservation areas, and its natural regeneration is good. However, it has minimal opportunities to move upwards under climate warming scenarios because it is on the highest mountain in Taiwan. In response to the impact of warming, research on the natural regeneration and artificial restoration of the upper edge of *A. kawakamii* should be strengthened in the future.

## Figures and Tables

**Table 1:** The current actual area of *A. kawakamii* forest at each altitude range.

Elevation Zone (m)	Area (ha)	% of Total Area
2200–2299	0	0.0%
2300–2399	8	0.0%
2400–2499	26	0.2%
2500–2599	118	0.7%
2600–2699	284	1.7%
2700–2799	501	3.0%
2800–2899	986	5.9%
2900–2999	1874	11.1%
3000–3099	3262	19.4%
3100–3199	3736	22.2%
3200–3299	2770	16.4%
3300–3399	1666	9.9%
3400–3499	951	5.6%
3500–3599	489	2.9%
3600–3699	168	1.0%
3700–3799	15	0.1%
3800–3899	0	0.0%
Total	16,857	100.0%

**Table 4:** Under the +0.5~+2.0 °C warming scenario, the loss and gain of *A. kawakamii* potential distributions in Taiwan.

Scenario	Actual Distribution (16,857 ha)	Potential Distribution (73,151 ha)
+0.5 °C	Low loss: 3991 ha Medium loss: 0 ha High loss: 0 ha The total area of loss: 3991 ha (23.7%)	Low loss: 35,368 ha Medium loss: 0 ha High loss: 0 ha The total area of loss: 35,368 ha (48.3%) Low gain: 276 ha
+1.0 °C	Low loss: 7704 ha Medium loss: 2153 ha High loss: 0 ha Total area of loss: 9857 ha (58.5%)	Low loss: 33,644 ha Medium loss: 22,571 ha High loss: 0 ha Total area of loss: 56,216 ha (76.8%) Low gain: 580 ha
+1.5 °C	Low loss: 5925 ha Medium loss: 7102 ha High loss: 59 ha Total area of loss: 13,086 ha (77.6%)	Low loss: 13,238 ha Medium loss: 50,624 ha High loss: 185 ha Total area of loss: 64,047 ha (87.6%) Low gain: 545 ha
+2.0 °C	Low loss: 2923 ha Medium loss: 4588 ha High loss: 8709 ha Total area of loss: 16,220 ha (96.2%)	Low loss: 4992 ha Medium loss: 25,904 ha High loss: 40,282 ha Total area of loss: 71,178 ha (97.3%) Low gain: 475 ha

## Data Availability

Not applicable.

## Supplementary Materials

The following supporting information can be downloaded at: https://www.mdpi.com/article/10.3390/plants11101346/s1, Table S1: List of the 62 environmental variables; Table S2: The pairwise Pearson correlation coefficients of 21 pre-selected environmental variables.
