# Supplementary material for: Spatial Distribution and Climate Warming Impact on Abies kawakamii Forest on a Subtropical Island
Source: Plants (Basel). 2022 May 19;11(10):1346. doi: 10.3390/plants11101346 (PMC9146738; doi:10.3390/plants11101346)
Supplement: Supplementary file 1 [file plants-11-01346-s001.zip › plants-1718179-Supplementary.pdf]

Table S1. List of the 62 environmental variables of the study.

Code	Description of variable	Resource or reference
T1	mean January temperature	Chiu et al. (2009)
T2	mean February temperature	Chiu et al. (2009)
T3	mean March temperature	Chiu et al. (2009)
T4	mean April temperature	Chiu et al. (2009)
T5	mean May temperature	Chiu et al. (2009)
T6	mean June temperature	Chiu et al. (2009)
T7	mean July temperature	Chiu et al. (2009)
T8	mean August temperature	Chiu et al. (2009)
T9	mean September temperature	Chiu et al. (2009)
T10	mean October temperature	Chiu et al. (2009)
T11	mean November temperature	Chiu et al. (2009)
T12	mean December temperature	Chiu et al. (2009)
P1	January precipitation (= Bio14)	Chiu et al. (2009)
P2	February precipitation	Chiu et al. (2009)
P3	March precipitation	Chiu et al. (2009)
P4	April precipitation	Chiu et al. (2009)
P5	May precipitation	Chiu et al. (2009)
P6	June precipitation	Chiu et al. (2009)
P7	July precipitation	Chiu et al. (2009)
P8	August precipitation	Chiu et al. (2009)
P9	September precipitation (= Bio13)	Chiu et al. (2009)
P10	October precipitation	Chiu et al. (2009)
P11	November precipitation	Chiu et al. (2009)
P12	December precipitation	Chiu et al. (2009)
Bio1	annual mean temperature	Fick & Hijmans (2017)
Bio4	temperature seasonality	Fick & Hijmans (2017)
Bio5	max temperature of warmest month	Fick & Hijmans (2017)
Bio6	min temperature of coldest month	Fick & Hijmans (2017)
Bio8	mean temperature of wettest quarter	Fick & Hijmans (2017)
Bio9	mean temperature of driest quarter (= Bio11)	Fick & Hijmans (2017)
Bio10	mean temperature of warmest quarter	Fick & Hijmans (2017)

Table S1 (continued). List of the 62 environmental variables of the study.

Code	Description of variable	Resource or reference
Bio12	annual precipitation	Fick & Hijmans (2017)
Bio15	precipitation of seasonality	Fick & Hijmans (2017)
Bio16	precipitation of wettest quarter	Fick & Hijmans (2017)
Bio17	precipitation of driest quarter (= Bio19)	Fick & Hijmans (2017)
Bio18	precipitation of warmest quarter	Fick & Hijmans (2017)
BT	biotemperature	Holdridge (1967)
PER	potential evapotranspiration ratio	Holdridge (1967)
CI	coldness index	Kira (1991)
WI	warmth index	Kira (1991)
EWI	effective warmth index	Chiu et al. (2012)
HI	humidity index	Xu (1985)
PS	summer half-year precipitation	Su (1985)
PW	winter half-year precipitation	Su (1985)
PSR	ratio of PS to Bio12	Su (1985)
PWR	ration of PW to Bio12	Su (1985)
TAR	temperature annual range (≈ Bio7)	Tuhkanen (1980)
ELE	elevation above sea level	Forestry Bureau
SLO	slope inclination	Forestry Bureau
LAT	latitude of raster	Forestry Bureau
LON	longitude of raster	Forestry Bureau
SR	solar radiation	Forestry Bureau
DIS	dissection in a continuous raster	Evans (2011)
ROU	roughness in a continuous raster	Evans (2011)
SRR	surface relief ratio	Evans (2011)
CUR	surface curvature index	Evans (2011)
SP	slope position	Evans (2011)
SAR	surface/area ratio	Evans (2011)
CTI	compound topographic index	Evans (2011)
HLI	heat load index	Evans (2011)
TRAI	topographic radiation aspect index	Evans (2011)
WLS	whole light sky space	Lai et al. (2010)

REFERENCES

Chiu CA, Lin PH, Lu KC. (2009). GIS-based tests for quality control of meteorological data and spatial interpolation of climatic data: A case study in mountainous Taiwan. Mountain Research and Development 29(4): 339-349.

Chiu CA, Lin, PH, Hsu CK, Shen ZH. (2012). A novel thermal index improves prediction of vegetation zones: Associating temperature sum with thermal seasonality. Ecological Indicators 23: 668-674.

Evans J. (2011). Geomorphometry and gradient metrics toolbox. Available at: http://conserveonline.org/workspaces/emt/documents/arcgis-geomorphometrics-toolbox/view.html. Accessed in: August 8th 2014.

Fick SE, Hijmans RJ. (2017). WorldClim 2: New 1-km spatial resolution climate surfaces for global land areas. International journal of climatology 37(12): 4302-4315.

Holdridge LR. (1967). Life zone ecology. Tropical Science Center, 148 p.

Kira T. (1991). Forest ecosystems of east and southeast Asia in a global perspective. Ecological Research 6(2): 185-200.

Lai YJ, Chou MD, Lin PH. (2010). Parameterization of topographic effect on surface solar radiation. Journal of Geophysical Research 115(D1): D01104.

SU HJ. (1985). Studies on the climate and vegetation types of the natural forests in Taiwan (3): A scheme of geographical climatic regions. Quarterly Journal of Chinese Forestry 18(3): 33-44.

Table S2. The pairwise Pearson correlation coefficients of 21 pre-selected environmental variables.

	ASP	SR	CTI	TRAI	WLS	bio1	bio4	bio10	bio11	T1	T7	EWI	bio12	bio15	bio18	bio19	PS	PW	PSR	PWR	PER
ASP		-0.027	-0.195	0.361	-0.100	-0.073	-0.066	-0.077	-0.065	-0.066	-0.078	-0.072	0.099	-0.028	0.104	0.028	0.129	0.019	-0.007	0.006	-0.138
SR	-0.027		0.151	0.476	0.476	-0.154	-0.049	-0.155	-0.161	-0.164	-0.157	-0.154	-0.099	0.016	-0.024	-0.043	-0.059	-0.083	0.071	-0.076	0.028
CTI	-0.195	0.151		0.013	0.403	0.467	0.373	0.480	0.427	0.423	0.478	0.465	-0.464	0.245	-0.229	-0.221	-0.406	-0.267	0.224	-0.230	0.598
TRAI	0.361	0.476	0.013		0.087	0.025	-0.032	0.014	0.030	0.029	0.012	0.026	-0.022	0.079	0.062	-0.058	0.035	-0.063	0.078	-0.078	0.026
WLS	-0.100	0.476	0.403	0.087		0.494	0.383	0.508	0.456	0.452	0.505	0.492	-0.396	0.231	-0.177	-0.162	-0.350	-0.224	0.203	-0.213	0.536
bio1	-0.073	-0.154	0.467	0.025	0.494		0.463	0.985	0.987	0.986	0.981	1.000	-0.429	0.449	-0.065	-0.312	-0.303	-0.314	0.320	-0.319	0.735
bio4	-0.066	-0.049	0.373	-0.032	0.383	0.463		0.604	0.321	0.320	0.618	0.450	-0.336	-0.302	-0.698	0.287	-0.692	0.171	-0.295	0.269	0.527
bio10	-0.077	-0.155	0.480	0.014	0.508	0.985	0.604		0.949	0.948	1.000	0.983	-0.429	0.322	-0.201	-0.206	-0.402	-0.222	0.202	-0.205	0.744
bio11	-0.065	-0.161	0.427	0.030	0.456	0.987	0.321	0.949		1.000	0.942	0.990	-0.386	0.510	0.042	-0.366	-0.204	-0.344	0.370	-0.363	0.682
T1	-0.066	-0.164	0.423	0.029	0.452	0.986	0.320	0.948	1.000		0.942	0.988	-0.376	0.497	0.033	-0.356	-0.207	-0.328	0.351	-0.344	0.675
T7	-0.078	-0.157	0.478	0.012	0.505	0.981	0.618	1.000	0.942	0.942		0.978	-0.424	0.298	-0.224	-0.187	-0.416	-0.202	0.177	-0.180	0.739

EWI	-0.072	-0.154	0.465	0.026	0.492	1.000	0.450	0.983	0.990	0.988	0.978		-0.428	0.457	-0.054	-0.319	-0.294	-0.320	0.328	-0.327	0.733
bio12	0.099	-0.099	-0.464	-0.022	-0.396	-0.429	-0.336	-0.429	-0.386	-0.376	-0.424	-0.428		-0.378	0.334	0.671	0.688	0.747	-0.523	0.524	-0.861
bio15	-0.028	0.016	0.245	0.079	0.231	0.449	-0.302	0.322	0.510	0.497	0.298	0.457	-0.378		0.586	-0.782	0.241	-0.742	0.887	-0.868	0.436
bio18	0.104	-0.024	-0.229	0.062	-0.177	-0.065	-0.698	-0.201	0.042	0.033	-0.224	-0.054	0.334	0.586		-0.372	0.901	-0.366	0.604	-0.597	-0.346
bio19	0.028	-0.043	-0.221	-0.058	-0.162	-0.312	0.287	-0.206	-0.366	-0.356	-0.187	-0.319	0.671	-0.782	-0.372		-0.010	0.933	-0.851	0.825	-0.500
PS	0.129	-0.059	-0.406	0.035	-0.350	-0.303	-0.692	-0.402	-0.204	-0.207	-0.416	-0.294	0.688	0.241	0.901	-0.010		0.032	0.223	-0.216	-0.674
PW	0.019	-0.083	-0.267	-0.063	-0.224	-0.314	0.171	-0.222	-0.344	-0.328	-0.202	-0.320	0.747	-0.742	-0.366	0.933	0.032		-0.925	0.920	-0.568
PSR	-0.007	0.071	0.224	0.078	0.203	0.320	-0.295	0.202	0.370	0.351	0.177	0.328	-0.523	0.887	0.604	-0.851	0.223	-0.925		-0.998	0.454
PWR	0.006	-0.076	-0.230	-0.078	-0.213	-0.319	0.269	-0.205	-0.363	-0.344	-0.180	-0.327	0.524	-0.868	-0.597	0.825	-0.216	0.920	-0.998		-0.458
PER	-0.138	0.028	0.598	0.026	0.536	0.735	0.527	0.744	0.682	0.675	0.739	0.733	-0.861	0.436	-0.346	-0.500	-0.674	-0.568	0.454	-0.458	
